# Extraction of High Quality DNA from Seized Moroccan Cannabis Resin (Hashish)

**DOI:** 10.1371/journal.pone.0074714

**Published:** 2013-10-04

**Authors:** Moulay Abdelaziz El Alaoui, Marouane Melloul, Sanaâ Alaoui Amine, Hamid Stambouli, Aziz El Bouri, Abdelmajid Soulaymani, Elmostafa El Fahime

**Affiliations:** 1 Functional Genomic Platform, UATRS, Center for Scientific and Technical Research [CNRST], Rabat, Morocco; 2 Laboratory of Forensic Sciences of Moroccan Gendarmerie Royale [LARATES], Rabat, Morocco; 3 Laboratoire de Génétique et Biométrie, Département de Biologie, Faculté des Sciences, Université Ibn Tofaïl, Kénitra, Maroc; Natural History Museum of Denmark, Denmark

## Abstract

The extraction and purification of nucleic acids is the first step in most molecular biology analysis techniques. The objective of this work is to obtain highly purified nucleic acids derived from *Cannabis sativa* resin seizure in order to conduct a DNA typing method for the individualization of cannabis resin samples. To obtain highly purified nucleic acids from cannabis resin (*Hashish*) free from contaminants that cause inhibition of PCR reaction, we have tested two protocols: the CTAB protocol of Wagner and a CTAB protocol described by Somma (2004) adapted for difficult matrix. We obtained high quality genomic DNA from 8 cannabis resin seizures using the adapted protocol. DNA extracted by the Wagner CTAB protocol failed to give polymerase chain reaction (PCR) amplification of tetrahydrocannabinolic acid (THCA) synthase coding gene. However, the extracted DNA by the second protocol permits amplification of THCA synthase coding gene using different sets of primers as assessed by PCR. We describe here for the first time the possibility of DNA extraction from (*Hashish*) resin derived from *Cannabis sativa*. This allows the use of DNA molecular tests under special forensic circumstances.

## Introduction

*Hashish* is the Arabic word for a particular form of resin made from *Cannabis sativa* [1]. *Hashish* has been historically used to describe all sorts of cannabis concoctions; today the word is primarily used to refer to cannabis resin. Derivatives of cannabis include: (i) **Cannabis herb (Marihuana):** Marihuana generally refers to the dried flowers and leaves of the cannabis plant. (ii) **Cannabis resin (** ***hashish*** **):** is the dried brown or black resinous secretion of the flowering tops of the cannabis plant. (iii) **Cannabis oil:** also called Hash oil, is a liquid made from combining *hashish* or the marihuana “bud” with solvents such as isopropyl alcohol.

When the plant is flowering, a ‘miniature glands called trichomes’ produces a resinous sap that concentrates a large part of the cannabinoids. There are two ways of collecting cannabis resin: **(i) Hand Rubbing :** is the oldest method of collection and preparation of trichomes, dried plants are gently rubbed until resin glands are excreted onto the hands and fingers. (ii) **Sieving:** Plants are first dried, the resin and trichomes become dusty and more brittle and can be separated from plant material using a sieve and a percussive force.

**Figure 1:**
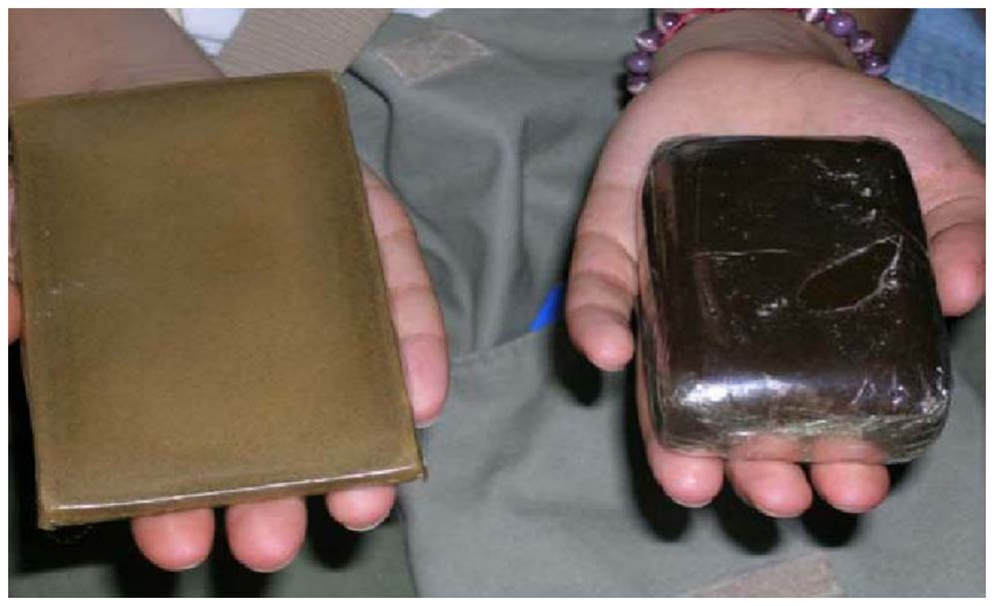
Example of compressed cannabis resin (Hashish) confiscated by Moroccan customs. Left hand: heated block of Hashish. Right hand: unheated block of Hashish.

The purest cannabis resin is obtained with light tapping, but larger amounts of plant material can be collected by increasing the force applied, the produced resin powder is heated gently, either pressed manually or mechanically to make it malleable (Figure 1).

There are several qualities of cannabis resin, which vary depending on the country of origin and the method of the preparation. Cannabis resin made from the first sifting is high grade resin, as it contains the maximum amount of resin and fewer impurities.

The cannabis resin can range in colour from blonde to brown to black. Its consistency can vary from modelling clay to brittle consistency, and these differences can be attributed to: (**i**) the variety of the cannabis plant used, (**ii**) the way in which it was grown and conserved, (**iii**) the presence of non-resinous plant material, (**iv**) how much the resin was pressed, heated or manipulated, (**v**) the age of resin, (**vi**) the adulterants introduced by drug dealers [2].

In Morocco, one of the main difficulties faced by law enforcement agencies controlling illicit drugs, in particular the *Cannabis sativa* derivatives, is the great uncertainty against the geographical origin of cannabis resin seizures. The relationship between chemical analysis and geographical origin of Moroccan *Cannabis sativa* has been described in previous studies [3]; however, current researchers are trying to find an analytical method other than a chemical assay to examine and classify samples seized [4]. DNA analysis can play an important role in the identification and discrimination between *Cannabis sativa* species [5]–[7]. In this context, DNA analysis test will be used to study the genetic variability of *Cannabis sativa* species using codominant short tandem repeat (STR) markers [8].

During seizure operations conducted by Moroccan customs the extraction of DNA from seized cannabis resin will help to determine its genetic profile and therefore its geographic origin. For example a sample S1 originated from a region R1 maybe seized in regions R2, R3 or R4.

The same DNA profile obtained will help to establish the connection traffic pathways, the linking source (region R1) and the tracking distribution networks (region R2, R3, R4).

Hence, the need to develop a reliable protocol for extraction of highly purified nucleic acids derived from the *Cannabis sativa* resin (*Hashish*), which is the most common form of seizure in checkpoints.

The Medicinal plants are known for high contents of secondary metabolites rich in polyphenols and/or polysaccharides [9]–[12]. High contents of polyphenolics and polysaccharides in plant leaves are problematic during the isolation of high-quality/quantity intact genomic nucleic acids [11], [13]. The presence of these contaminants in DNA preparations often makes the samples viscous and renders DNA unrestrictable in endonuclease digestion and unamplifiable in PCR [14].

DNA extraction methods can vary depending on the complexity of the plant material and matrix used. Researchers have developed different protocols for genomic DNA extraction from leaves, seeds/grains and processed food/feed samples [15].

The cetyltrimethylammonium bromide (CTAB) method is the most used for DNA extraction from plants. The method was first developed by Murray and Thompson [16] and was modified later by Wagner [17] There are several modifications of the CTAB protocol that involve the addition of proteinase K and mercaptoethanol to the CTAB buffer and the use of phenol/chloroform/isoamyl alcohol (25∶24∶1) initially and then chloroform/isoamyl alcohol (24∶1) for further purification [18], [19]. There are also differences in protocols regarding the concentrations of the CTAB extraction buffer components used.

Extraction of DNA from leaf, flower, and seed samples of Cannabis sativa has been reported previously [20]. To our knowledge, this is the first paper describing the extraction of DNA from cannabis resin (Hashish) considered as a very complex matrix. In this study, we have tested two protocols: the CTAB protocol of Wagner [17] and a CTAB protocol adapted for complex matrix [21]. In this study, we investigate the possibility to extract DNA from cannabis resin starting with the hypothesis that Wagner CTAB protocol used routinely in our laboratory to extract DNA from plants such as the Rosa damascena, and Vigna unguiculata will also extract DNA from Hashish since it comprising glandular trichomes (cannabinoids production factory) ; these trichomes contain a secretory cell disk and therefore the genetic information which is DNA.

## Materials and Methods

### Samples

Eight samples of cannabis resin (*Hashish*) seizure were provided by the *Laboratory of Forensic Sciences of Moroccan Gendarmerie Royal* [LARATES]. These samples were seized from the North of Morocco.

### DNA extractions

We have tested two protocols for DNA extraction from the eight cannabis *Hashish* samples. A Wagner CTAB genomic DNA isolation method [17] originated from the research of Murray and Thompson and Somma CTAB protocol adapted for GMO in food matrix [21]. The only parameter we changed in the adapted protocol described by Somma is the weight of the matrix used, 200 mg instead of 100 mg. All the others steps are kept as described in the protocol.

### Yield and quality of nucleic acid extracts

DNA extracted by both protocols was quantified using NanoDrop 8000 spectrophotometer (Thermo). One absorbance unit (260 nm) was assumed to correspond to 50 ng of nucleic acid per µl of solution. The purity of the samples was estimated from the (A_260_/A_280_) ratio.

### Repurification step

Since DNA extracted by the Wagner CTAB protocol was of poor quality, a re-purification step was necessary. The ethanol precipitation is used to concentrate and precipitating DNA in the presence of ammonium acetate as binding salt [22].

### PCR amplification and detection

To check the suitability of extracted DNA using both protocols, a downstream analysis by PCR reaction was performed. To this end, we have chosen to amplify for the full-length coding region of the THC synthase gene using the following primers a/b as described previously [23](Table 1), and the amplification of different fragments of the gene using a combination of primers g/f; a/f; c/e; c/h; d/h; d/b (Table 1). The same combination of primers was used for sequencing in order to assemble the complete sequence of the THCA synthase gene. The Amplification was performed in a total volume of 25 µl, with each reaction containing, 1× reaction buffer (Invitrogen), 2.5 mMgCl2, 0.5× Q-Solution (Qiagen), 0.2 mM dNTPs, 0.4 µM of each primers, 0.04 U/µl of Platinum DNA polymerase (Invitrogen), and 20 ng/µl of template DNA. The PCR conditions were: preheating at 94°C for 5 min, 35 cycles at 94°C for 1 min, 55°C for 1 min, and 72°C for 2 min with a final extension at 72°C for 10 min. The reactions were carried out in a BIO RAD S1000 Thermal Cycler.

**Table 1:** Sequences of oligonucleotides used in this study.

Primer	Sequence	Reference
a	5′-TGAAGAAAAAAAATGAATTGCTCAGCATTTTCC-3′	[23]
b	5′-TCTATTTAAAGATAATTAATGATGATGCGGTGG-3′	
c	5′-CAAACTGGTTGCTGTCCCATC-3′	
d	5′-AGCTGGGAAGAAGACGGCTTTCTCA-3′	
e	5′-CGTCTTCTTCCCAGCTGATCT-3′	
f	5′-CGCCAACAGTAGGGCAATACC-3′	
g	5′-AATAACTCCCATATCCAAGCA-3′	
h	5′-AGGACTCGCATGATTAGTTT-3′	

### Agarose gel electrophoresis

The amplified fragments were electrophoresed on 1% agarose gel and detected using ethidium bromide along with a molecular weight marker 1 kb (Invitrogen). The DNAs integrity of all samples extracted with both protocols was assessed using 0.8% agarose gel electrophoresis. The gels were visualised under UV trans-illuminator using the G: BOX gel documentation system (Syngene).

### Sequence analysis

The amplified PCR products were purified using ExoSAP-IT PCR Purification Kit (Affymetrix) according to the manufacturer's instructions. For each purified PCR product, we have performed a sequencing reaction with the forward and reverse primer a/b; g/f; a/f; c/e; c/h; d/h ;d/b. Sequencing reaction was carried out using the BigDye Terminator cycle Sequencing Kit v3.1 (Applied Biosystems) according to the manufacturers' instructions. Automated DNA sequencing was performed using an Applied Biosystems 3130XL sequencer. Sequence data were assembled using DNA Baser Sequence Assembler [24].

### Nucleotide sequence accession numbers

The THC synthase gene sequences determined in the present study has been deposited under the GenBank accession numbers: JQ437481–JQ437488.

## Results and Discussion

### Yield and quality of nucleic acid extracts

DNA extraction using Wagner CTAB protocol provided a yield ranging from 15.3 to 373.8 (ng/µl).This yield was about 40.88 to 303.4 (ng/µl) after an additional step of purification (Table 2).

**Table 2:** DNA yield and purity of isolated DNA from the eight cannabis resin samples using Wagner and adapted CTAB protocol (all DNA were re-dissolve in 100 µl sterile deionised water).

	Wagner CTAB protocol before re-purification step						Wagner CTAB protocol after repurification step						Adapted CTAB protocol					
samples	A230	A260	A280	A260/230	A260/280	ng/µl	A230	A260	A280	A260/230	A260/280	ng/µl	A230	A260	A280	A260/230	A260/280	ng/µl
1	1,86	0,31	0,26	0,57	1,17	15,30	0,53	2,39	1,28	1,26	1,87	119,60	1,37	1,63	0,86	2,24	1,91	81,57
2	0,11	7,20	4,84	0,81	1,49	359,90	0,49	2,44	1,30	1,20	1,88	122,10	0,51	4,49	2,18	2,30	2,06	224,30
3	0,30	2,90	1,92	0,87	1,51	145,00	0,39	2,53	1,53	0,99	1,65	126,50	0,80	2,95	1,42	2,37	2,07	147,60
4	0,19	4,90	2,89	0,92	1,69	245,10	0,30	3,08	1,79	0,91	1,72	153,80	4,35	0,64	0,30	2,79	2,15	32,06
5	1,85	0,31	0,24	0,58	1,30	15,68	0,19	6,07	3,37	1,17	1,80	303,30	0,77	3,02	1,54	2,33	1,96	151,20
6	0,10	7,48	4,97	0,72	1,50	373,80	0,36	2,59	1,50	0,93	1,72	129,30	1,90	1,24	0,62	2,35	1,99	61,88
7	0,28	2,42	1,63	0,67	1,49	121,20	0,31	2,26	1,51	0,69	1,49	112,80	3,85	0,48	0,23	1,85	2,13	24,00
8	0,12	4,65	7,07	0,56	0,66	232,60	2,25	0,82	0,49	1,84	1,66	40,88	0,27	8,48	4,44	2,28	1,91	424,20

With the Adapted CTAB protocol the yield was 24 to 424.2(ng/µl) (Table 2).

Concerning the quality of nucleic acids, Wagner CTAB protocol with an additional step of purification gives a value of the ratio (A_260_/A_280_) ranging from 1.49 to 1.88. This may be due to high levels of phenolic and polysaccharides in the samples as previously reported [25]. However this ratio (A_260_/A_280_) ranged from 1.9 to 2.15 with the second CTAB protocol, indicating high quality nucleic acids extracted. On the other hand, the ratio (A_260_/A_230_) was also used as a secondary measurement of nucleic acids purity. The standard value of this ratio (A_260_/A_230_) ranges from 2.0 to 2.2.The Adapted CTAB protocol gives the best values of the (A_260_/A_230_) ratio. However, this ratio was lower than 2 for all samples extracted by the Wagner CTAB protocol, even after an additional purification step (Table 2). This may indicate the presence of potential contaminants which absorb at 230 nm [26].

The differences observed in DNA quality is probably due to the major differences between the tow protocols used : the adapted CTAB protocol is successfully optimized and upgraded by including enzymatic digestion using proteinase K and RNase, the addition of the CTAB precipitation solution that remove additional possible polysaccharides and polyphenolic compound that might be combined with DNA and therefore affecting the purity and quality of the extracted DNAs, the incubation steps involved in this protocol to improve the DNA quality,and the addition of an overnight isopropanol precipitation step, also the ultracentrifugation speed and incubation time used can explain also the differences in quality and purity obtained.

DNAs extracted with Wagner CTAB protocol were of bad quality, these maybe indicate the presence of polysaccharides and polyphenolic compound, the presence of such kind of contaminant may interfere negatively during downstream reactions like PCR amplification or DNA digestion [14]. To test this hypothesis, we decided to amplify the coding region of the THC synthase gene by PCR using DNA extracted by both protocols as template.

### PCR amplification of THC synthase gene from different samples

The quality and integrity of DNA obtained by the two protocols was assessed by PCR amplification of full-length coding region of the THCA synthase gene using specific primers (a/b).

**Figure 2:**
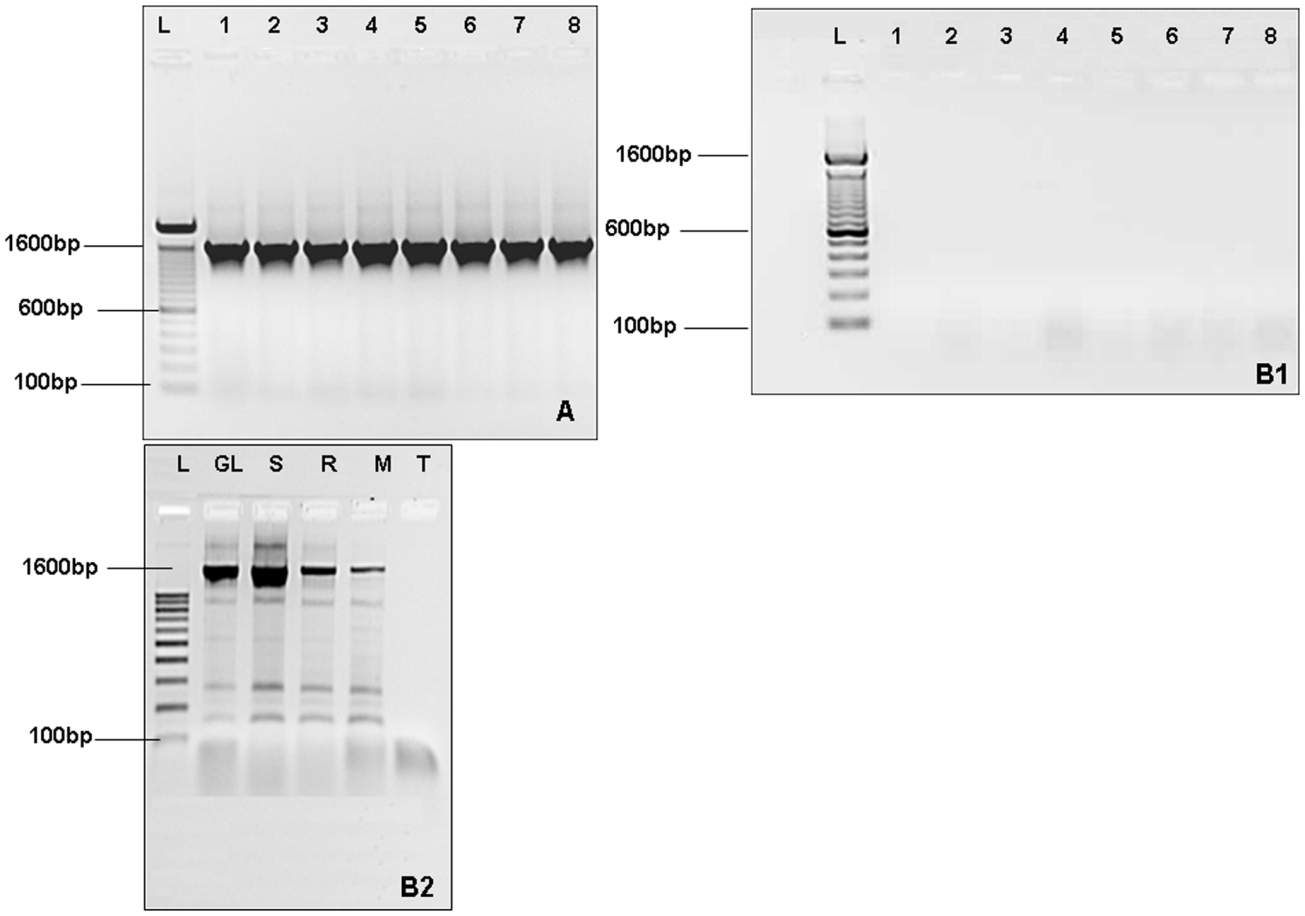
Agarose gel electrophoresis (1%) of PCR amplification product of THCA synthase gene using the primers (a/b). (A): Amplification of DNA extracted by the adapted protocol. (B1): Amplification of DNA extracted by Wagner CTAB protocol (cannabis resin). (1–8): Indicated number of samples; (L): 100 bp molecular size ladder; the position of 100 bp, 600 bp and 1600 bp fragments is indicated. (B2): Amplification of DNA extracted by Wagner CTAB protocol (using other tissues of *cannabis sativa*). (GL): Grind leaves of cannabis sativa. (S): Seeds of cannabis. (R): Rod of cannabis sativa. (M): Mixed rood and leaves. (T): Negative control.

The PCR amplification of THCA synthase was successful on DNA obtained by the Adapted CTAB protocol (Figure 2A). However, no amplification was obtained with DNA extracted by the Wagner protocol (Figure 2B1). This may be explained by the presence of PCR inhibitor in DNA extracted by the Wagner CTAB protocol, and/or by the loss of DNA integrity.

### Electrophoretic analysis of genomic DNA extracts

**Figure 3:**
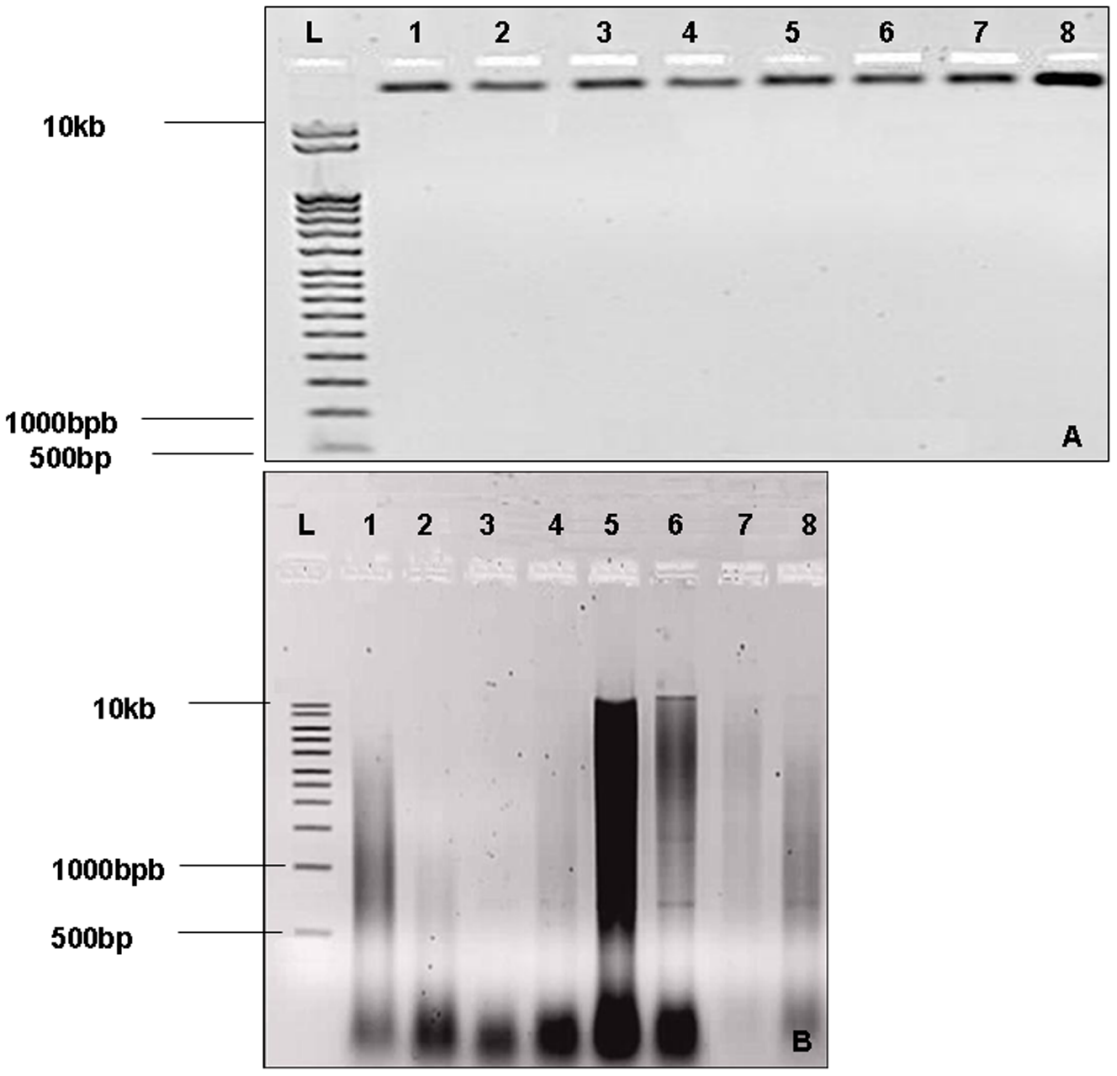
(A): Agarose gel analysis (0.8%) of extracted gDNA by the adapted protocol. (B) : Agarose gel analysis (0.8%) of extracted gDNA by the Wagner protocol. (1–8): Indicated number of samples; (L): 10 kb molecular size ladder with the position of 500 bp, 1000 bp and 10 kb fragments indicated.

DNA samples extracted with Wagner CTAB protocol showed smearing bands, which indicate that the DNAs were highly degraded and/or fragmented and may explain the PCR failure of THC synthase gene. (Figure 3B).

DNA samples extracted with the Adapted CTAB protocol show one predominant band in all samples (Figure 3A) indicating intact gDNA, and thus improved preservation of DNA integrity making it possible to perform amplification across the sequence of the gene of interest. This amplified DNA was further submitted to another downstream method test based on DNA sequencing.

### Sequencing

To assess the possibility of sequencing amplified DNAs, we have chosen to sequence the complete coding region of the THCA synthase gene in one sample (Sample1). Amplicons obtained by the combination of primers (mentioned above) that cover the total sequence were submitted to sequence analysis on the ABI genetic analyser. All electropherograms were of good quality. After the assembly of all sequence data, a complete sequence of the THCA synthase gene was generated.

These results demonstrate that this protocol was proven to be suitable for the extraction of high quality DNA from cannabis resin (*Hashish*) that could be used as a template in routine DNA sequencing techniques.

### Conclusion

Our study demonstrates that the Adapted CTAB protocol for difficult matrix allowed us to obtain a high quality DNA from cannabis resin samples. This DNA quality was sufficient to perform amplification of a specific gene using PCR technique. Wagner CTAB protocol is largely used to extract genomic DNA from different plants, but it is not ideal in the case of cannabis resin. In our experiments, the adapted protocol was used in routine extraction of DNA from cannabis resin. The high DNA quality obtained permit downstream DNA analysis techniques such as PCR and sequencing. These techniques will contribute to the identification of the geographic origin of seized cannabis resin (Hashish) conducted by the police or by Customs in checkpoints and therefore establish the connection traffic pathways.

Hence the necessity of finding a reliable protocol for the extraction of highly purified nucleic acids derived from *Cannabis sativa* resin seizure which is the most common form of seizure in checkpoints.

Developing such kind of DNA extraction from unusual material derived from cannabis plant (like *Hashish*) will be of great interest given its close association with forensic casework.
